# Augmented Modality Exclusivity Norms for Concrete and Abstract Italian Property Words

**DOI:** 10.5334/joc.88

**Published:** 2019-10-24

**Authors:** Piermatteo Morucci, Roberto Bottini, Davide Crepaldi

**Affiliations:** 1 Basque Center on Cognition, Brain and Language (BCBL), Donostia, ES; 2 Centre for Mind/Brain Sciences, University of Trento, IT; 3 International School for Advanced Studies (SISSA), Trieste, IT

**Keywords:** Word processing, Semantics, Stimulus development, Embodied cognition

## Abstract

How perceptual information is encoded into language and conceptual knowledge is a debated topic in cognitive (neuro)science. We present modality norms for 643 Italian adjectives, which referred to one of the five perceptual modalities or were abstract. Overall, words were rated as mostly connected to the visual modality and least connected to the olfactory and gustatory modality. We found that words associated to visual and auditory experience were more unimodal compared to words associated to other sensory modalities. A principal components analysis highlighted a strong coupling between gustatory and olfactory information in word meaning, and the tendency of words referring to tactile experience to also include information from the visual dimension. Abstract words were found to encode only marginal perceptual information, mostly from visual and auditory experience. The modality norms were augmented with corpus–based (e.g., Zipf Frequency, Orthographic Levenshtein Distance 20) and ratings–based psycholinguistic variables (Age of Acquisition, Familiarity, Contextual Availability). Split-half correlations performed for each experimental variable and comparisons with similar databases confirmed that our norms are highly reliable. This database thus provides a new important tool for investigating the interplay between language, perception and cognition.

## Introduction

The meaning of words has been suggested to be grounded, at least partially, in the perceptual and motor system (Meteyard et al., 2012, Barsalou et al., 2008). This idea, usually referred to as embodied semantics (Barsalou, 1999; Glenberg & Gallese, 2012) has found support from many neuroimaging studies showing that processing word meaning involves the recruitment of modality-specific networks distributed across the cortex (Barsalou, 2008; Binder & Desai, 2011; Vigliocco et al. 2009). For instance, processing words associated with auditory features (e.g., “telephone”) activates auditory areas more than processing words associated with visual features (e.g., “moon”; Kiefer et al., 2008). Similarly, words referring to concepts that can be experienced through different senses (e.g., “football”) activate multiple modality-specific networks (i.e., visual and action–related; Van Dam et al., 2012). According to the embodied framework, sensory-motor areas directly contribute to language processing via situated simulation, that is, a partial re-enactment of the neural activity crucial for perceptual, motor and affective experience (Connell & Lynott, 2012).

In order to study the link between sensorimotor experience and conceptual processing it is important to have tools that allow a precise characterization of the sensorimotor characteristics usually associated with a given word/concept. For instance, is a word heavily based on perceptual information? Is it more strongly associated to vision or touch? Is it unimodal (e.g., only visual: “red”) or multimodal (e.g., visuo-haptic: “round”)?

The modality exclusivity norms developed by Lynott and Connell (2009, 2013; see also Lynott et al., 2019, for the largest existing dataset) provide an elegant tool to inspect the perceptual structure of concepts, and allow researchers to select property words based on specific sensory features of interest. These norms are created by asking subjects to rate the extent to which a word can be experienced through each of the five senses (vision, audition, touch, taste, smell). By averaging the ratings in each modality across subjects, each word is represented as a vector where each value reflects the strength of perceptual information for each sensory domain. Mean sensory ratings are then typically used to compute further metrics tracking, e.g., modality exclusivity and maximal perceptual strength (Lynott & Connell, 2009). These metrics sum up the individual scores on each sense and provide a general characterization of the words’ perceptual load profile.

The development of these norms has had an impact in the field. For instance, maximum perceptual strength (i.e., perceptual strength in the dominant modality) has been shown to consistently outperform both concreteness and imageability ratings in accounting for variance in response latency and accuracy in lexical decision, word naming and memory tasks (Connell & Lynott, 2012; van Dantzig et al., 2011). These norms have been also important to highlight some recurrent interactions between the perceptual structure and lexical characteristics of words. For instance, Lynott and Connell (2013) showed that some aspect of surface word form (e.g., word length, frequency) are associated with the perceptual information that is encoded in the corresponding concept.

We believe that these norms may be beneficial for several other experimental purposes. For example, they might be a valuable tool to improve stimuli selection in studies investigating the relationship between the perceptual organization of concepts and semantic categories (e.g., food, colors, tools; Gainotti et al., 2013), to model behavioral performance in computational simulation, or to study how perceptual information is integrated into holistic conceptual representations at the neural level (Clarke & Tyler, 2014; Martin et al., 2018). They might be also important for cross–linguistic studies investigating the extent to which the perceptual organization of concepts is based on culturally specific vs. culturally independent factors (Majid et al., 2018; Majid & Burenhult, 2014). Finally, they may help to improve experimental design in studies investigating the role of perceptual experience in conceptual processing with children (e.g., Della Rosa et al., 2010), atypical and sensory deprived populations (e.g., blind, deaf; e.g., Bedny et al., 2019), even in conjunction with other larger datasets including nouns and verbs (Vergallito et al., 2019).

To aid researchers working in these areas, we provide modality exclusivity norms for 643 Italian property words. Property words (i.e., adjectives) are the class of words that arguably better denotes the sensory content of perceptual experience (e.g., red, bright, perfumed, soft; Winter et al., 2018), as well as the class that exhibits higher modality exclusivity (i.e., the class where it is easier to find purely unimodal words; Lynott & Connell, 2013). This would favor the production of highly-controlled stimulus sets, allowing to uncover more subtle effects related to the perceptual content of word meaning. In addition to a mean score for each perceptual modality, we report for each word a series of general indexes that track the overall perceptual profile (e.g., modality exclusivity, maximal perceptual strength; Lynott and Connell, 2009). Differently from most of the existing similar datasets, (i) we included both concrete and abstract words (to the best of our knowledge, only Lynott et al., 2019 has the same feature); and (ii) we augmented the database with a number of psycholinguistic covariates, by collecting further ratings (for Age of Acquisition, AoA, Familiarity, Fam, and Contextual Availability, CA; Carroll & White, 1973; Gernsbacher, 1984; Schwanenflugel & Shoben, 1983) and by computing objective metrics, such as frequency and number of orthographic neighbors (N), based on other existing databases (Subtlex–IT; Crepaldi et al., 2016).

## Methods

### Participants

A total of 383 native Italian speakers (254 females) participated in this study; 202 (148 females) took up the perceptual strength questionnaire while 181 (106 females) rated words for Fam, AoA, and CA. They were aged 19 to 68 years (mean = 27.22; SD = 10.25). Subjects were recruited and performed the study on line, via Google Forms.

### Materials

We created a list of 643 Italian property words (i.e., adjectives). We tried to include adjectives referring to all five sensory modalities (e.g., vision: *rosso*, red; audition: *rumoroso*, loud; touch: *liscio*, smooth; taste: *delizioso*, delicious; smell: *profumato*, perfumed), as well as highly multimodal (e.g., *forte*, strong) and abstract concepts (e.g., *puro*, pure). Words were collected from a wide range of sources. Where possible, we translated lexical items from Lynott and Connell’s database (2009).

### Procedure

The full set of 643 words was randomly split into five lists/surveys (three lists of 129 items and two lists of 128 items). Each survey was rated by 40 to 42 subjects. Participants were presented with each word separately and were asked to rate the extent to which they usually experience the meaning of that word through each of the five senses (touch, audition, vision, smell, and taste). The scale spanned from 0 (not at all) to 5 (greatly). The instructions given to the subjects were translated from Lynott & Connell (2009). Participants were told that once they had rated a word in each of the five modalities, they could move to the next word. Participants were also told that there were no right or wrong answers, and were encouraged to use their own judgments. Finally, participants were instructed to skip a word when they did not know its meaning. Each questionnaire took about 15–20 minutes to complete.

A similar procedure was adopted to collect ratings for Fam, AoA, and CA. For each variable, the full set of words was randomly split into three surveys (two of 214 and one of 215 items), for a total of 9 surveys. Twenty to twenty-one subjects rated each survey. Familiarity ratings were acquired by asking participants to rate words on a 7-point scale according to their frequency of occurrence in everyday life. The rating scale went from one (not at all familiar/frequent) to seven (extremely familiar/frequent). The instructions to participants were based on Gernsbacher (1984). For AoA, participants were asked for the age at which they think they learnt a word (Carroll & White, 1973), similarly to previous studies (Ghyselinck et al., 2000; Kuperman et al., 2012; Montefinese et al., 2019). Because some previous research on AoA used age bands rather than continuous estimates (e.g., Barca et al., 2002), numeric values were converted into 7-point scale values. The scale ranges from 1 (age 0–2 years) to 7 (age 13 years and older), with intermediate points on the scale corresponding to 2-year age bands. CA ratings were collected by asking people to rate words on a 7-point scale according to how easy it is to find a context or circumstance in which a word might be used. The rating scale went from one (context not at all available) to seven (context extremely available). The instructions to participants were taken from Schwanenflugel and Stowe (1989).

For each word we also obtained objective frequency (measured in Zipf; Van Heuven et al., 2014; or normalised on a million words), Orthographic Neighborhood Size (N; Coltheart et al., 1977), the mean orthographic Levenshtein distance to the 20 closest neighbours (OLD20; Yarkoni et al., 2008), contextual diversity (CD, the number of contexts in a corpus in which a word appears; Adelman et al., 2006) and word length in letters. These metrics are all based on the Subtlex-IT corpus (Crepaldi et al., 2016). We report these variables in the database along with the modality norms. Forty-two words were not present in Subtlex-IT and thus present missing values for these objective variables.

Following Lynott and Connell (2009), the database was augmented with a few variables that characterize each word’s perceptual profile: *mean modality score*, the mean ratings of how strongly a word is experienced through each of the five senses; *dominant modality*, the modality with the higher strength value; *maximum perceptual strength*, the strength in the dominant modality; and *modality exclusivity*, which tracks the extent to which a particular concept is perceived through a single modality, and is computed as the range of the perceptual scores across the five modalities divided by summed perceptual strength (a purely unimodal word will have a modality exclusivity of 1, while a completely multimodal will have a value of 0). We also report the overall *sum of perceptual strength values*, the sum of the values related to the five perceptual modalities. Finally, we computed *entropy* across the perceptual scores, which tracks again their variability across the five sense (e.g., entropy is minimal when one value is very high and the other four are very low, i.e., in strongly unimodal words; and maximal when all values are rather similar, i.e., in strongly multimodal words).

Because (i) different groups of subjects rated different word lists and (ii) subjects may have used the scale quite differently, all metrics are offered both raw and based on standardized scores, which were obtained through a z transformation operated within subject:

{\hat S_{ij}} = \frac{{{S_{ij}} - E\left( {{S_j}} \right)}}{{\sigma \left( {{S_j}} \right)}}

where S_ij_ is the response given by subject *j* to word *i*, and E(S_j_) and σ(S_j_) are the overall mean and standard deviation for subject *j*.

## Results and discussion

As a first step, we cleaned our datasets from unreliable responses. Fourteen participants were excluded because they completed less than 60% of the survey (nine, one, three and one subject for perceptual strength, Fam, CA and AoA ratings respectively).

Additional unreliable participants were captured through the clustering procedure illustrated in Rodriguez and Laio (2014). Subjects were modeled as points in an N–dimensional space, where N equals the number of judgments that each participant offered. The ratings for each word define the position of each participant/point in this space, so that participants with similar judgments will be close and participants with different judgments will be relatively far apart. By applying a clustering algorithm to this space, it is possible to identify outliers as unclustered datapoints. Rodriguez and Laio’s procedure was applied separately for each questionnaire and variable, and led to the exclusion of four subjects for what concerns perceptual strength ratings; and five, one and five subjects for Fam, CA and AoA ratings, respectively.

Overall, we collected 130,080, 13,074, 12,860 and 12,860 datapoints for perceptual strength, AoA, Fam and CA, respectively; of these, we discarded 8,370 (6.4%), 1,287 (9.8%), 1,285 (10%), and 857 (6.7%).

We will illustrate in what follows the reliability of the metrics included in the database, and their distribution across the words included therein. In addition, we will describe the perceptual structure of the Italian lexicon as it emerges in the database, via univariate and correlational analyses.

### Reliability of the measures

**Figure 1:**
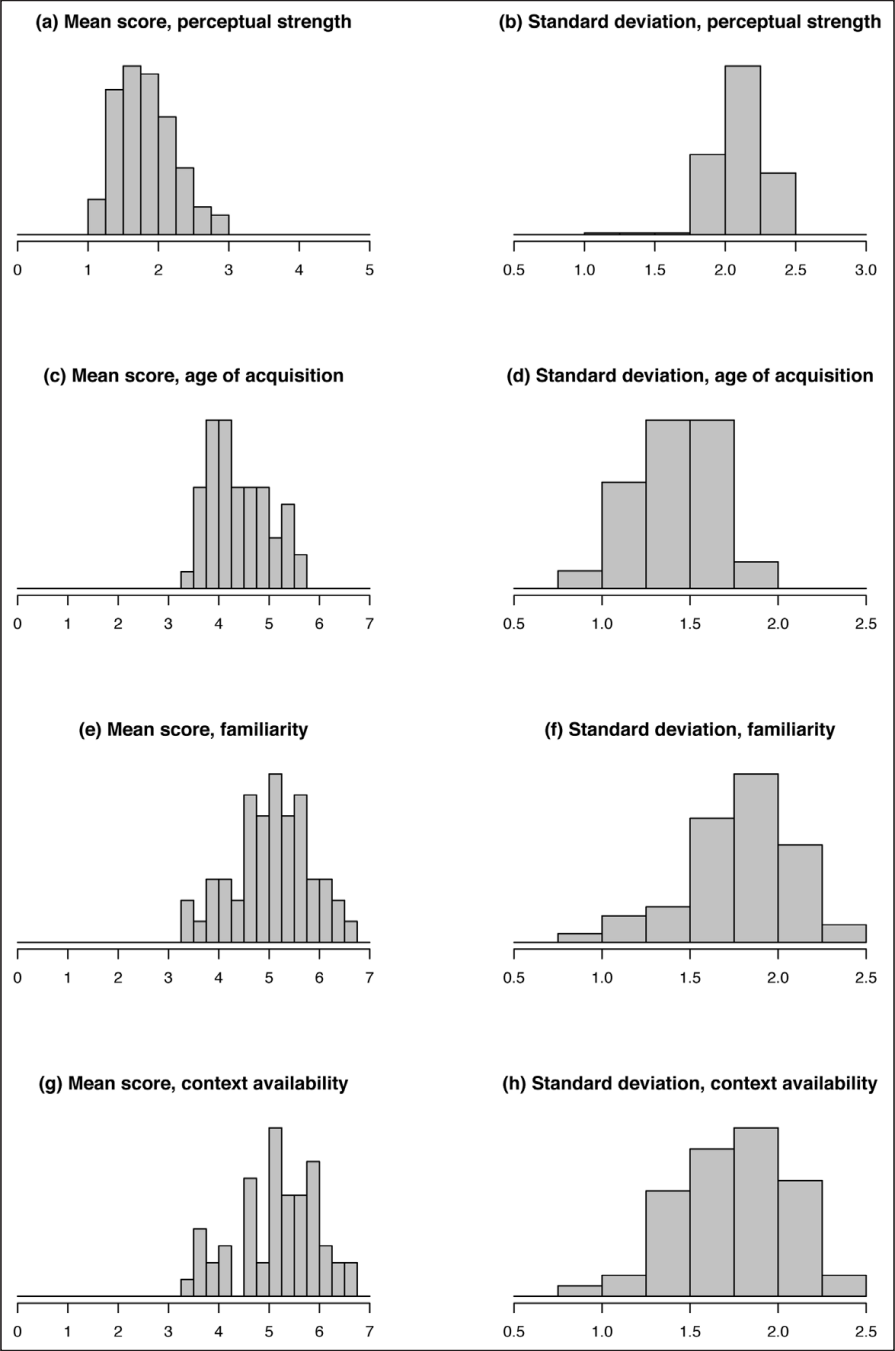
Distributions of each subject’s overall mean score and standard deviation, for each of the ratings collected in this study.

We first checked how our participants used the response scales (Figure 1). There are interesting differences (e.g., participants cluster more towards the upper end of the Fam and CA scales, as compared to perceptual strength and AoA), but overall, participants seem to have interpreted the scales rather consistently on all variables. This is reflected in the very high correlation between raw and standardized ratings (0.998, 0.988, .986 and 0.992, for perceptual strength, AoA, Fam and CA, respectively).

**Figure 2:**
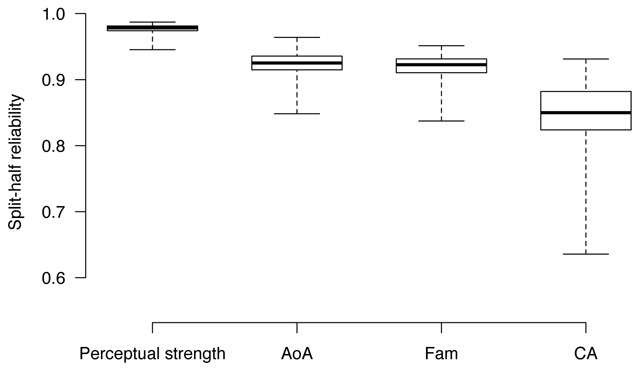
Boxplots illustrating the distributions of the split–half reliability scores over 1,000 random replicates.

Reliability was checked by computing split–half correlations over 1,000 random replicates, separately for each questionnaire. Results are reported in Figure 2, and show good reliability for all ratings—all median correlations are well above 0.80, with a peak 0.98 for perceptual strength judgments. CA fares worse than the other variables, particularly in terms of its lower distribution tail; this mirrors some previous results showing more variability in CA judgments as compared to other lexical, questionnaire–based variables (Borelli et al., 2018), and may be related to the fact that CA depends more on individual experience, or is perhaps too broadly defined to elicit consistent judgements across participants.

We also compared the ratings produced by our participants against those collected in previous similar studies. We found a strong correlation (r = 0.92) between our sensory ratings and those collected for the corresponding English translations by Lynott and Connell (2009), suggesting that the perceptual profile of words generalizes quite well between English and Italian. We also attempted to assess the external reliability of the other psycholinguistic variables that we gathered in this new database (Fam, AoA and CA) against those of other existing Italian datasets. Most of the resources available for Italian focus on nouns (e.g., Della Rosa et al., 2010; Navarrete et al., 2019), and those that include adjectives are typically rather specific (e.g., Borelli et al., 2018, took up pain words). Therefore, we found only a limited number of shared words to test our metrics. Yet, correlations were all very strong, especially for AoA (r = 0.93 on the 84 words in common with Montefinese et al., 2019) and Fam (r = 0.92 on the 37 words in common with Borelli et al., 2018). The correlation coefficient was slightly weaker for CA, but still solid (r = 0.79 on the 37 words in common with Borelli eta al., 2018). Together with the split-half correlations presented above, these correlational patterns confirm the high reliability of our new metrics.

### Perceptual structure of the database

#### Descriptive statistics

**Figure 3:**
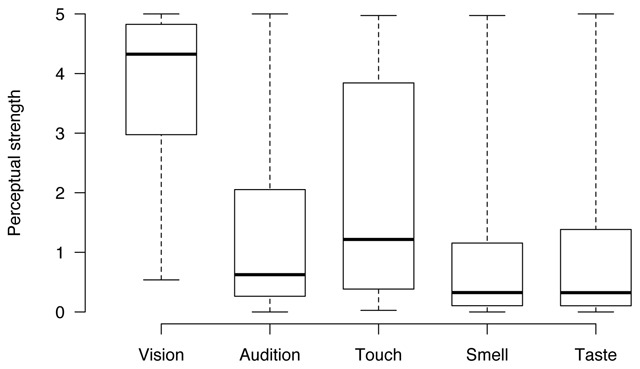
Distributions of the perceptual strength scores on the five senses.

The perceptual profile of the words included in our database is illustrated in Table 1 and Figure 3. Overall, words in our database were judged to be experienced mostly in the visual modality. Perhaps a bit more surprisingly, the second strongest modality is touch, while audition elicited only slightly higher ratings than smell and taste. All modalities range very widely, essentially over the whole scale; this indicates that our database includes words across the entire perceptual strength space, quite nicely.

**Table 1:** Perceptual strength across the five modalities for the words included in the database.

Modality	Mean	SD	Min	Max

Visual	3.83	1.17	0.54	5.00
Auditory	1.33	1.44	0	5.00
Haptic	1.95	1.71	0.03	4.97
Gustatory	0.98	1.37	0	5.00
Olfactory	0.94	1.28	0	4.97

Vision is also the dominant modality for the majority of the words in our database (N = 413), followed by audition and touch (N = 86 and N = 74, respectively). Words dominated by taste or smell are the least common (N = 46 and N = 24, respectively). This pattern is very similar to what was found in previous studies with English (Lynott and Connell, 2009) and Dutch (Speed and Majid, 2017). However, it slightly differs from previous work in Mandarin Chinese, where auditory words were found to be the less common together with olfactory words (Chen et al., 2019). The large distribution of visual information across words in our database is consistent with previous work on other languages like English (Lynott & Connell, 2009), Dutch (Speed and Majid, 2017), Russian (Miklashevsky, 2018), Mandarin Chinese (Chen et al., 2019) and Serbian (Đurđević et al., 2016). Overall, this crosslinguistic pattern provides support for the hypothesis that language semantics exhibits visual dominance in vocabulary usage and structure (Levinson & Majid, 2014; San Roque et al., 2015).

**Figure 4:**
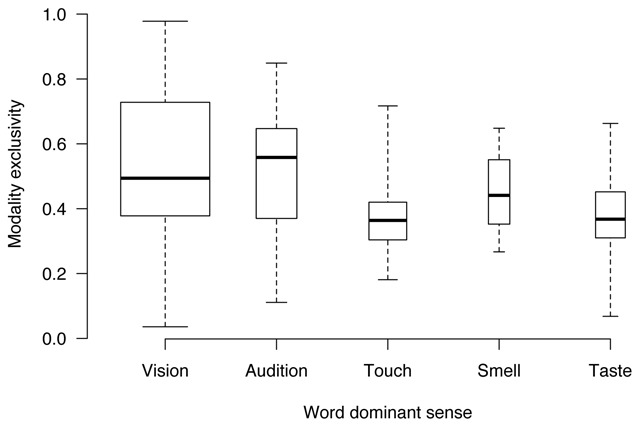
Distributions of modality exclusivity scores, computed separately for words with a different dominant sense. Box width is proportional to the number of words that fall into each of the five categories.

As illustrated in Figure 4, visual and auditory words span modality exclusivity quite widely, indicating that the database include both strongly unimodal and strongly multimodal visual and auditory words. Smell words seem to cluster more at the centre of the distribution, while taste and touch words extends a bit more widely, and reach further down the scale towards multimodality (taste words in particular). Despite these differences in uni/multimodality, the average strength in the dominant sense is quite similar in visual (4.41), auditory (4.05), haptic (4.54), olfactory (4.33) and gustatory words (4.69). This seems to suggest that the database features strong words in all modalities, but words strong on touch, smell or taste tend to be also strong on vision and audition (that is, they tend to be multimodal). These patterns are not surprising, since words referring to smell and taste usually refer to highly multimodal concepts such as flavors, drinks and food (e.g., *delizioso*, delicious; *dolce*, sweet). On the other hand, many words dominated by vision in our dataset refer to color (e.g., *rosso*, red; *nero*, black), which indeed can be only experienced through vision. Similarly, many words referring to the auditory modality describe aspects of our experience that cannot be accessed through other senses (e.g., *melodioso*, melodic; *rumoroso*, noisy), thus making them more unimodal compared to words referring to taste, touch or smell.

**Figure 5:**
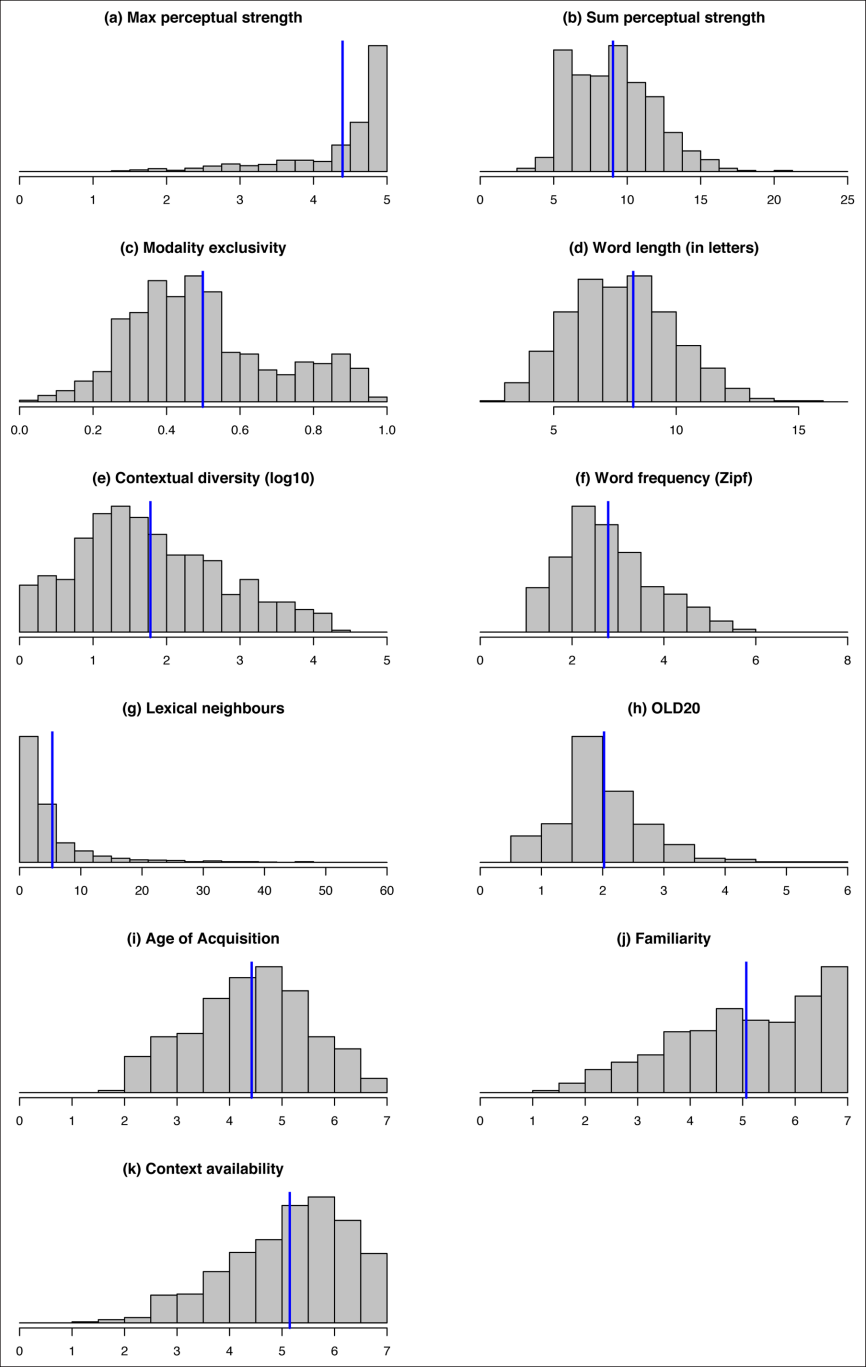
Density plots illustrating the distributions of the main lexical and perceptual strength features of the words included in the database. The blue vertical line represents the mean.

Figure 5 illustrates the features of the words included in the database more generally. Max perceptual strength is strongly right–tailed; because the database focuses on perceptual words, this was to be expected. Note, though, that the lower tail is also populated—these are the abstract words that we included in this database to contrast perceptual words. If we consider the (unavoidably arbitrary) cut–off adopted by Connell et al., (2018), that is, 2.9, we have 48 abstract words. The perceptual information that was attributed to these words is mainly visual (the median score on vision is 2.34, Q1 = 1.91, Q3 = 2.67) and auditory (M = 2.00, Q1 = 1.56, Q3 = 2.30). Unsurprisingly, their modality exclusivity is very low (M = 0.35, Q1 = 0.10, Q3 = 0.54) compared to the remaining concrete words (M = 0.48, Q1 = 0.03, Q3 = 0.98), as no modality considerably dominates over the others. The other variable distributions are less tailed, with the exception of number of lexical neighbors (most words in the database live in a rather sparse lexical space). AoA, Fam and CA distributions are nicely wide, but they do tend more towards the upper end—this was also expected, given that concrete words tend to be acquired early, be rather familiar and easily elicit contexts of use (Paivio, 1971; Schwanenflugel, 1991).

**Figure 6:**
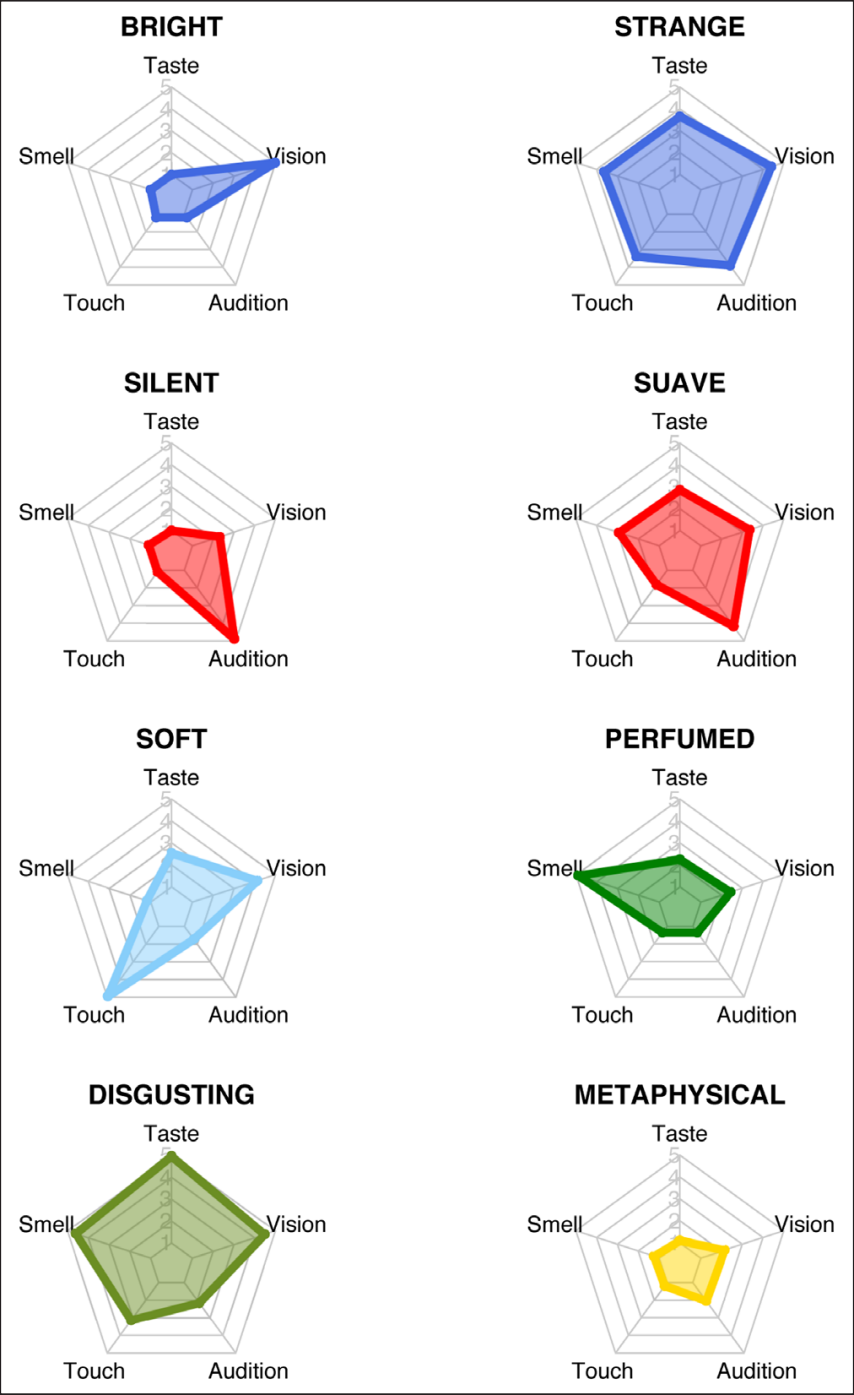
Spider plots illustrating the perceptual strength scores on the five senses for some example words, each of which is deemed representative of its class (vision unimodal for “bright”, vision multimodal for “strange”, audition unimodal for “silent”, audition multimodal for “suave”, touch for “soft”, smell for “perfumed”, taste for “disgusting” and abstract for “metaphysical”).

Figure 6 offers a few spider plots illustrating with example words the properties that we just highlighted.

#### Correlational analyses

Table 2 illustrates the correlation between the perceptual variables and the other lexical–semantic indexes (e.g., Zipf, AoA). The top-left area of the correlational matrix reports correlations among the perceptual variables, and is dominated by the strong relationship between gustatory and olfactory ratings (r = 0.76). This pattern replicates a finding that emerged in previous similar studies using English, (Lynott and Connell, 2009), Dutch (Speed & Majid, 2017), Mandarin Chinese (Chen et al., 2019) and Russian (Miklashevsky, 2018), uncovering an interesting cross–linguistic symmetry between Italian and these languages. The coupling of gustatory and olfactory experience in language semantics fits well within theories claiming that flavor perception can be considered as a unified, multisensory sense (e.g., Auvray & Spence, 2008). The second strongest positive correlation is between haptic and visual ratings (r = 0.34). The tendency of touch words to include information from the visual dimension likely reflects the fact that these words usually define properties of objects and tools, which can indeed be typically accessed through both vision and touch. Auditory ratings negatively correlate with all the other ratings, showing a general tendency of auditory information to dissociate from information coming from other senses.

**Table 2:** Correlation matrix between all variables included in the database. Significant correlations are bolded (p < .001). Abbreviations refer to the following variables: Maximal Perceptual Strength (MPS), Summed Perceptual Strengths (SPS), Modality Exclusivity, Contextual Diversity (CD), Subtlex-IT Frequency (Zipf), Neighborhood Size (N), Orthographic Levenshtein Distance 20 (OLD20), Familiarity (Fam), Age of Acquisition (AoA), Context Availability (CA).

Variables	Vision	Audition	Touch	Olfaction	Taste	MPS	SPS	Entropy	ME	Length	CD	Zipf	N	OLD20	Fam	AoA	CA

Vision	–	**–0.46**	**0.34**	**–0.31**	**–0.3**	**0.48**	0.1	**–0.3**	**0.25**								
Audition	**–0.46**	–	**–0.22**	**–0.16**	**–0.18**	**–0.24**	0.02	**0.19**	**–0.21**								
Touch	**0.34**	**–0.22**	–	–0.12	0.04	**0.19**	**0.61**	**0.35**	**–0.46**								
Olfaction	**–0.31**	**–0.16**	–0.12	–	**0.76**	–0.04	**0.55**	**0.52**	**–0.46**								
Taste	**–0.3**	**–0.18**	0.04	**0.76**	–	0	**0.64**	**0.54**	**–0.51**								
MPS	**0.48**	**–0.24**	**0.19**	–0.04	0	–	**0.17**	**–0.46**	**0.47**								
SPS	0.1	0.02	**0.61**	**0.55**	**0.64**	**0.17**	–	**0.7**	**–0.75**								
ME	**–0.3**	**0.19**	**0.35**	**0.52**	**0.54**	**–0.46**	**0.7**	–	**–0.96**								
Entropy	**0.25**	**–0.21**	**–0.46**	**–0.46**	**–0.51**	**0.47**	**–0.75**	**–0.96**	–								
Length	–0.05	0.08	–0.1	–0.06	–0.1	–0.06	–0.12	–0.07	0.05	**–**	**–0.28**	**–0.37**	**–0.6**	**0.78**	**–0.23**	**0.3**	**–0.21**
CD	0.05	**0.16**	0.06	0.04	0.01	–0.06	**0.16**	**0.19**	**–0.19**	**–0.28**	**–**	**0.68**	**0.54**	**–0.41**	**0.4**	**–0.46**	**0.31**
Zipf	0.08	**0.15**	0.1	0.06	0.07	–0.07	**0.23**	**0.26**	**–0.26**	**–0.37**	**0.68**	**–**	**0.52**	**–0.57**	**0.79**	**–0.64**	**0.67**
N	0.11	–0.01	0.09	0	0.04	0.04	0.12	0.07	–0.07	**–0.6**	**0.54**	**0.52**	**–**	**–0.63**	**0.38**	**–0.44**	**0.33**
OLD20	–0.04	–0.05	–0.11	–0.04	–0.11	0	**–0.19**	**–0.19**	**0.17**	**0.78**	**–0.41**	**–0.57**	**–0.63**	**–**	**–0.42**	**0.43**	**–0.38**
Fam	**0.13**	0.06	**0.14**	–0.04	0.07	–0.06	**0.19**	**0.2**	**–0.21**	**–0.23**	**0.4**	**0.79**	**0.38**	**–0.42**	**–**	**–0.66**	**0.85**
AoA	**–0.31**	0.04	**–0.2**	0.02	–0.06	**–0.4**	**–0.25**	0.05	–0.04	**0.3**	**–0.46**	**–0.64**	**–0.44**	**0.43**	**–0.66**	**–**	**–0.77**
CA	**0.26**	0.01	**0.22**	–0.02	0.11	**0.25**	**0.29**	0.08	–0.09	**–0.21**	**0.31**	**0.67**	**0.33**	**–0.38**	**0.85**	**–0.77**	**–**

Importantly, the coupling of gustatory and olfactory experience in language semantics, as well as the dominance of vision mentioned above, seem to hold not just across languages, but also across grammatical categories (e.g., adjectives, nouns, verbs). Indeed, despite our database includes only adjectives, we nicely replicate previous studies using nouns and verbs in this respect (e.g., Vergallito et al., 2019; Winter et al., 2018; Speed and Majid, 2017), which is interesting given that words belonging to different grammatical categories play different roles in language, and usually denote bunches of perceptual experience that differ quite importantly (e.g., objects, descriptions, events and activities).

The bottom-left part of the correlational matrix illustrates the relationship between the perceptual and the psycholinguistic variables. Maximal perceptual strength correlates negatively with AoA (r = –0.40) and CA (r = –0.25)—perceptually loaded words are typically learnt earlier in life and more easily elicit contexts of use (Paivio, 1971; Schwanenflugel, 1991). Interestingly, AoA correlates negatively also with visual (r = –0.31) and haptic (r = –0.20) scores, suggesting that the property words that are usually acquired earlier in life are those referring to visual and haptic experience.

**Figure 7:**
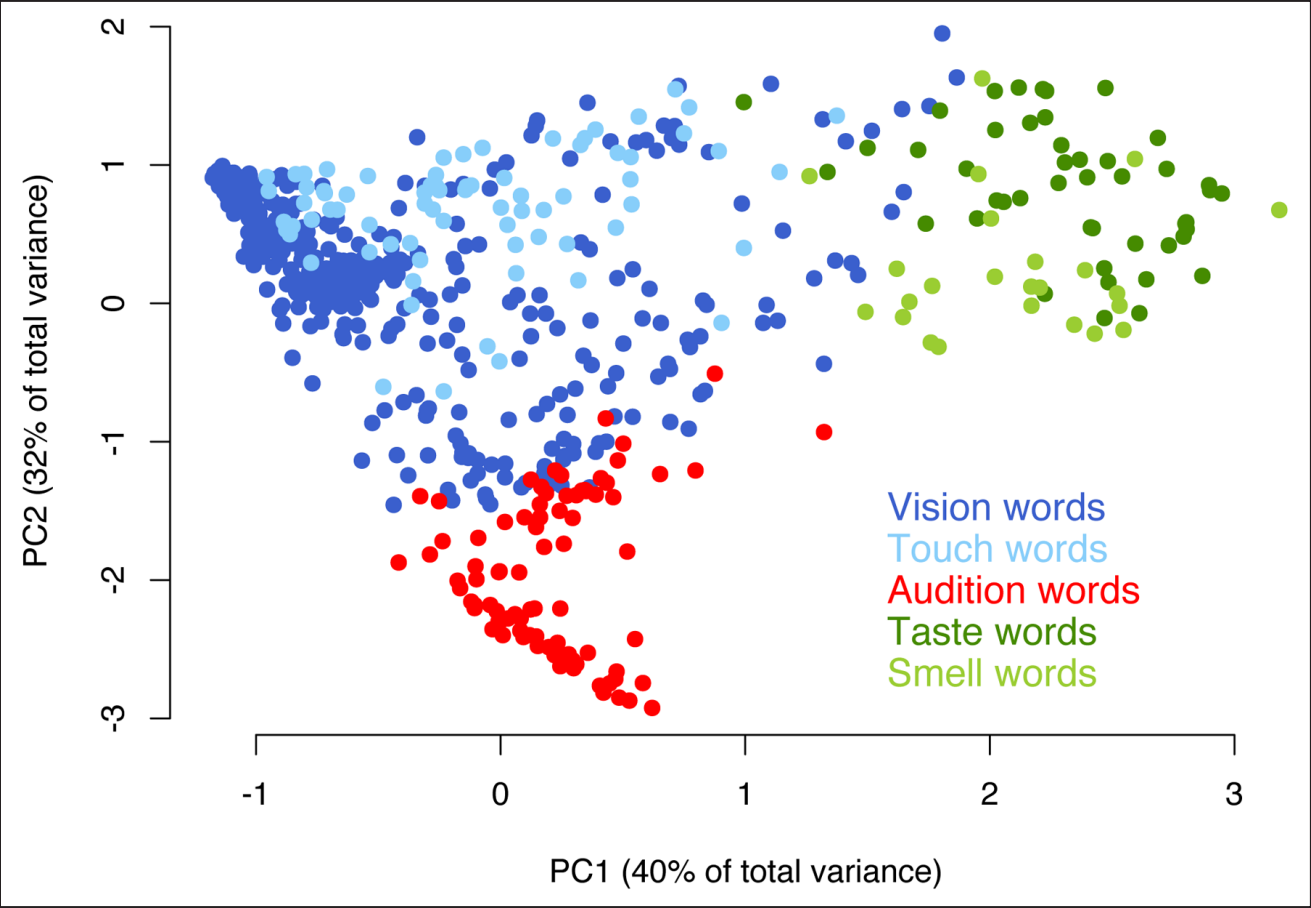
The words included in the database, as represented by points in the space defined by the first two Principal Components of the unrotated PCA.

**Figure 8:**
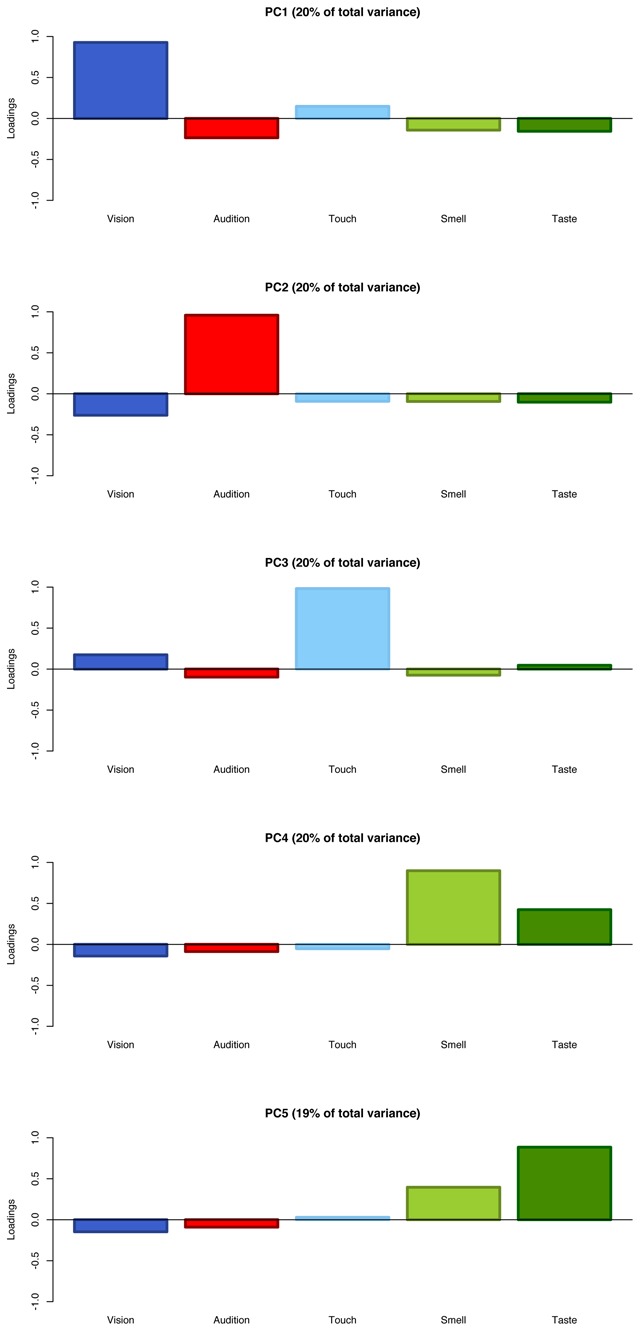
Loadings on the five modalities by five Principal Components when a Varimax rotation is applied to PCA to maximise component interpretability.

The perceptual structure behind our modality strength judgments was explored more in depth via Principal Component Analysis. The two most important dimensions account for 72% of the total variance and, in line with the correlational patterns, highlight a visuo–tactile, an auditory and a gustatory–olfactory cluster (Figure 7). Interestingly, when a Varimax rotation is applied to make the components more easily interpretable, each of them does seem to specifically track one sense (although this is less clear for smell and taste, as expected; Figure 8; Speed & Majid, 2017).

## Conclusion

We developed the first database with modality exclusivity norms for Italian words, as has previously been done with English (Lynott and Connell, 2009), Dutch (Speed and Majid, 2017), Serbian (Đurđević et al., 2016), Russian (Miklashevsky, 2018) and Chinese (Chen et al., 2019). The database includes 643 words, thus providing a wide set of stimuli to help researchers interested in the relationship between language and perception. For each word we computed a mean score on each sensory modality, plus a number of general metrics that track the overall perceptual profile of each word (e.g., Maximum Perceptual Strength, Modality Exclusivity). We also augmented the database with several subjective and objective lexical–semantic indexes that are known to influence word processing (e.g., Zipf frequency, AoA). This resource is thus much more than a mere set of modality exclusivity norms, and allows to tap perceptual variables against other lexical metrics unrelated to perception.

The database has a few key features that makes it an ideal tool for Psycholinguistic and Cognitive Neuroscience research. First, ratings are extremely reliable. This is shown by split–half correlations, the correlation with other existing databases and the very consistent use of the scale by our participants, which essentially makes subject–normalized scores redundant (they correlate nearly perfectly with the raw, uncorrected ratings). In addition to these nice statistical features, the database includes: (i) words whose ratings span very widely from essentially no perceptual load to very strong perceptual strength on all senses; (ii) dominant words for each of the five senses; (iii) both strongly unimodal and strongly multimodal words; (iv) abstract words. This means that researchers will find words with a perceptual profile as diverse as it can get.

Among other purposes, our norms can serve to improve experimental design in studies investigating the role of perceptual experience in semantic processing, even with children, atypical and sensory deprived populations; or in neuroimaging studies looking at how perceptual information is integrated into conceptual knowledge at the neural level. Further, they can be used in conjunction with existing datasets collected in other languages to investigate consistent vs. differential aspects of how languages code for perception.

## Data Accessibility Statement

All the materials related to this paper (the stimulus list, the data collected, the analysis script, and the tools that we used during the analysis) are publicly available at the Open Science Framework (https://osf.io/s8gv9/).

## References

[1] Adelman, J. S., Brown, G. D., & Quesada, J. F. (2006). Contextual diversity, not word frequency, determines word-naming and lexical decision times. Psychological science, 17(9), 814–823. DOI: 10.1111/j.1467-9280.2006.01787.x 16984300

[2] Auvray, M., & Spence, C. (2008). The multisensory perception of flavor. Consciousness and cognition, 17(3), 1016–1031. DOI: 10.1016/j.concog.2007.06.005 17689100

[3] Barca, L., Burani, C., & Arduino, L. S. (2002). Word naming times and psycholinguistic norms for Italian nouns. Behavior Research Methods, Instruments, & Computers, 34(3), 424–434. DOI: 10.3758/BF03195471 12395559

[4] Barsalou, L. W. (1999). Perceptual symbol systems. Behavioral and brain sciences, 22(4), 577–660. DOI: 10.1017/S0140525X99002149 11301525

[5] Barsalou, L. W. (2008). Grounded cognition. Annu. Rev. Psychol., 59, 617–645. DOI: 10.1146/annurev.psych.59.103006.093639 17705682

[6] Barsalou, L. W., Santos, A., Simmons, W. K., & Wilson, C. D. (2008). Language and simulation in conceptual processing. Symbols, embodiment, and meaning, 245–283. DOI: 10.1093/acprof:oso/9780199217274.003.0013

[7] Bedny, M., Koster-Hale, J., Elli, G., Yazzolino, L., & Saxe, R. (2019). There’s more to “sparkle” than meets the eye: Knowledge of vision and light verbs among congenitally blind and sighted individuals. Cognition, 189, 105–115. DOI: 10.1016/j.cognition.2019.03.017 30939375

[8] Binder, J. R., & Desai, R. H. (2011). The neurobiology of semantic memory. Trends in cognitive sciences, 15(11), 527–536. DOI: 10.1016/j.tics.2011.10.001 22001867 PMC3350748

[9] Borelli, E., Crepaldi, D., Porro, C. A., & Cacciari, C. (2018). The psycholinguistic and affective structure of words conveying pain. PloS one, 13(6), e0199658 DOI: 10.1371/journal.pone.0199658 29958269 PMC6025857

[10] Carroll, J. B., & White, M. N. (1973). Age-of-acquisition norms for 220 picturable nouns. Journal of Verbal Learning and Verbal Behavior, 12(5), 563–576. DOI: 10.1016/S0022-5371(73)80036-2

[11] Chen, I. H., Zhao, Q., Long, Y., Lu, Q., & Huang, C. R. (2019). Mandarin Chinese modality exclusivity norms. PloS one, 14(2), e0211336 DOI: 10.1371/journal.pone.0211336 30785906 PMC6382104

[12] Clarke, A., & Tyler, L. K. (2014). Object-specific semantic coding in human perirhinal cortex. Journal of Neuroscience, 34(14), 4766–4775. DOI: 10.1523/JNEUROSCI.2828-13.2014 24695697 PMC6802719

[13] Coltheart, M. (1977). Access to the internal lexicon. The psychology of reading.

[14] Connell, L., & Lynott, D. (2012). Strength of perceptual experience predicts word processing performance better than concreteness or imageability. Cognition, 125(3), 452–465. DOI: 10.1016/j.cognition.2012.07.010 22935248

[15] Connell, L., Lynott, D., & Banks, B. (2018). Interoception: the forgotten modality in perceptual grounding of abstract and concrete concepts. Philosophical Transactions of the Royal Society B: Biological Sciences, 373(1752), 20170143 DOI: 10.1098/rstb.2017.0143 PMC6015840 29915011

[16] Crepaldi, D., Amenta, S., Mandera, P., Keuleers, E., & Brysbaert, M. (2016). Frequency estimates from different registers explain different aspects of visual word recognition. International Meeting of the Psychonomic Society, Granada, Spain, 5–8 May http://crr.ugent.be/subtlex-it/

[17] Della Rosa, P. A., Catricalà, E., Vigliocco, G., & Cappa, S. F. (2010). Beyond the abstract—concrete dichotomy: mode of acquisition, concreteness, imageability, familiarity, age of acquisition, context availability, and abstractness norms for a set of 417 Italian words. Behavior research methods, 42(4), 1042–1048. DOI: 10.3758/BRM.42.4.1042 21139171

[18] Đurđević, D. F., Popović Stijačić, M., & Karapandžić, J. (2016). A quest for sources of perceptual richness: Several candidates. Studies in language and mind, 187–238.

[19] Gainotti, G., Spinelli, P., Scaricamazza, E., & Marra, C. (2013). The evaluation of sources of knowledge underlying different conceptual categories. Frontiers in human neuroscience, 7, 40 DOI: 10.3389/fnhum.2013.00040 23439453 PMC3578198

[20] Gernsbacher, M. A. (1984). Resolving 20 years of inconsistent interactions between lexical familiarity and orthography, concreteness, and polysemy. Journal of experimental psychology: General, 113(2), 256 DOI: 10.1037/0096-3445.113.2.256 6242753 PMC4311894

[21] Ghyselinck, M., De Moor, W., & Brysbaert, M. (2000). Age-of-acquisition ratings for 2816 Dutch four-and five-letter nouns. Psychologica Belgica, 40(2), 77–98.

[22] Glenberg, A. M., & Gallese, V. (2012). Action-based language: A theory of language acquisition, comprehension, and production. Cortex, 48(7), 905–922. DOI: 10.1016/j.cortex.2011.04.010 21601842

[23] Kiefer, M., Sim, E. J., Herrnberger, B., Grothe, J., & Hoenig, K. (2008). The sound of concepts: four markers for a link between auditory and conceptual brain systems. Journal of Neuroscience, 28(47), 12224–12230. DOI: 10.1523/JNEUROSCI.3579-08.2008 19020016 PMC6671691

[24] Kuperman, V., Stadthagen-Gonzalez, H., & Brysbaert, M. (2012). Age-of-acquisition ratings for 30,000 English words. Behavior research methods, 44(4), 978–990. DOI: 10.3758/s13428-012-0210-4 22581493

[25] Levinson, S. C., & Majid, A. (2014). Differential ineffability and the senses. Mind & Language, 29(4), 407–427. DOI: 10.1111/mila.12057

[26] Lynott, D., & Connell, L. (2009). Modality exclusivity norms for 423 object properties. Behavior Research Methods, 41(2), 558–564. DOI: 10.3758/BRM.41.2.558 19363198

[27] Lynott, D., & Connell, L. (2013). Modality exclusivity norms for 400 nouns: The relationship between perceptual experience and surface word form. Behavior research methods, 45(2), 516–526. DOI: 10.3758/s13428-012-0267-0 23055172

[28] Lynott, D., Connell, L., Brysbaert, M., Brand, J., & Carney, J. (2019, 5 7). The Lancaster Sensorimotor Norms: Multidimensional measures of Perceptual and Action Strength for 40,000 English words. DOI: 10.31234/osf.io/ktjwp PMC7280349 31832879

[29] Majid, A., & Burenhult, N. (2014). Odors are expressible in language, as long as you speak the right language. Cognition, 130(2), 266–270. DOI: 10.1016/j.cognition.2013.11.004 24355816

[30] Majid, A., Roberts, S. G., Cilissen, L., Emmorey, K., Nicodemus, B., O’grady, L., …, Shayan, S. (2018). Differential coding of perception in the world’s languages. Proceedings of the National Academy of Sciences, 115(45), 11369–11376. DOI: 10.1073/pnas.1720419115 PMC6233065 30397135

[31] Martin, C. B., Douglas, D., Newsome, R. N., Man, L. L., & Barense, M. D. (2018). Integrative and distinctive coding of visual and conceptual object features in the ventral visual stream. Elife, 7, e31873 DOI: 10.7554/eLife.31873 29393853 PMC5832413

[32] Meteyard, L., Cuadrado, S. R., Bahrami, B., & Vigliocco, G. (2012). Coming of age: A review of embodiment and the neuroscience of semantics. Cortex, 48(7), 788–804. DOI: 10.1016/j.cortex.2010.11.002 21163473

[33] Miklashevsky, A. (2018). Perceptual Experience Norms for 506 Russian Nouns: Modality Rating, Spatial Localization, Manipulability, Imageability and Other Variables. Journal of psycholinguistic research, 47(3), 641–661. DOI: 10.1007/s10936-017-9548-1 29282595

[34] Montefinese, M., Vinson, D., Vigliocco, G., & Ambrosini, E. (2019). Italian age of acquisition norms for a large set of words (ItAoA). Frontiers in psychology, 10, 278 DOI: 10.3389/fpsyg.2019.00278 30814969 PMC6381031

[35] Navarrete, E., Arcara, G., Mondini, S., & Penolazzi, B. (2019). Italian norms and naming latencies for 357 high quality color images. PloS one, 14(2), e0209524 DOI: 10.1371/journal.pone.0209524 30794543 PMC6386297

[36] Paivio, A. (1971). Imagery and verbal processes. New York: Holt, Rinehart and Winston.

[37] Rodriguez, A., & Laio, A. (2014). Clustering by fast search and find of density peaks. Science, 344(6191), 1492–1496. DOI: 10.1126/science.1242072 24970081

[38] San Roque, L., Kendrick, K. H., Norcliffe, E., Brown, P., Defina, R., Dingemanse, M., …, Majid, A. (2015). Vision verbs dominate in conversation across cultures, but the ranking of non-visual verbs varies. Cognitive Linguistics, 26, 31–60.

[39] Schwanenflugel, P. J. (1991). Why are abstract concepts hard to understand? In P. J. Schwanenflugel (Ed.), The psychology of word meanings (pp. 223–250). Hillsdale, NJ: Lawrence Erlbaum Associates.

[40] Schwanenflugel, P. J., & Shoben, E. J. (1983). Differential context effects in the comprehension of abstract and concrete verbal materials. Journal of Experimental Psychology: Learning, Memory, and Cognition, 9(1), 82 DOI: 10.1037/0278-7393.9.1.82

[41] Speed, L. J., & Majid, A. (2017). Dutch modality exclusivity norms: Simulating perceptual modality in space. Behavior research methods, 49(6), 2204–2218. DOI: 10.3758/s13428-017-0852-3 28155185

[42] Van Dam, W. O., van Dijk, M., Bekkering, H., & Rueschemeyer, S. A. (2012). Flexibility in embodied lexical-semantic representations. Human brain mapping, 33(10), 2322–2333. DOI: 10.1002/hbm.21365 21976384 PMC6869997

[43] Van Dantzig, S., Cowell, R. A., Zeelenberg, R., & Pecher, D. (2011). A sharp image or a sharp knife: Norms for the modality-exclusivity of 774 concept-property items. Behavior Research Methods, 43(1), 145–154. DOI: 10.3758/s13428-010-0038-8 21287109 PMC3048290

[44] Van Heuven, W. J., Mandera, P., Keuleers, E., & Brysbaert, M. (2014). SUBTLEX-UK: A new and improved word frequency database for British English. The Quarterly Journal of Experimental Psychology, 67(6), 1176–1190. DOI: 10.1080/17470218.2013.850521 24417251

[45] Vergallito, A., Petilli, M. A., & Marelli, M. (2019, 6 11). Perceptual strength norms in Italian. Retrieved from osf.io/d6tjh

[46] Vigliocco, G., Meteyard, L., Andrews, M., & Kousta, S. (2009). Toward a theory of semantic representation. Language and Cognition, 1(2), 219–247. DOI: 10.1515/LANGCOG.2009.011

[47] Winter, B., Perlman, M., & Majid, A. (2018). Vision dominates in perceptual language: English sensory vocabulary is optimized for usage. Cognition, 179, 213–220. DOI: 10.1016/j.cognition.2018.05.008 29966914

[48] Yarkoni, T., Balota, D., & Yap, M. (2008). Moving beyond Coltheart’s N: A new measure of orthographic similarity. Psychonomic Bulletin & Review, 15(5), 971–979. DOI: 10.3758/PBR.15.5.971 18926991

